# Oxidative Stress, Vascular Remodeling, and Vascular Inflammation in Hypertension

**DOI:** 10.1155/2013/710136

**Published:** 2013-10-21

**Authors:** Nicolas Federico Renna

**Affiliations:** ^1^Laboratory of Cardiovascular Physiopathology, IMBECU-CONICET, Department of Pathology, School of Medicine, UNCuyo, 5500 Mendoza, Argentina; ^2^Institute of Experimental Medicine and Biology of Cuyo (IMBECU), CONICET, Mendoza, Argentina

Hypertension is a major public health problem worldwide, affecting over 50 million individuals in the United States alone. The modern history of hypertension begins with the understanding of the cardiovascular system with the work of the physician William Harvey (1578–1657), who described the circulation of blood in his book *De Motu Cordis*. The English clergyman Stephen Hales made the first published measurement of blood pressure in 1733. Frederick Akbar Mahomed (1849–1884) made the first report of elevated blood pressure in a person without evidence of kidney disease. However, hypertension as a clinical entity came into being in 1896 with the invention of the cuff-based sphygmomanometer by Scipione Riva-Rocci. This apparatus has allowed the measurement of the blood pressure into the clinic. In 1905, Nikolai Korotkoff improved the technique by describing the Korotkoff sounds that are heard when the artery is auscultated with a stethoscope while the sphygmomanometer cuff is deflated.

The concept of essential hypertension (“hypertonic essential”) was introduced in 1925 by the physiologist Otto Frank to describe elevated blood pressure for which no cause could be found. Over the several decades, increasing evidence accumulated from actuarial reports and longitudinal studies, such as the Framingham Heart Study. In the actuality, the term “inflammation” is used in the context of cardiovascular disease as a catchall referring to nonspecific phenomena, such as elevation of C-reactive protein. Most clinicians and investigators find this vague and difficult to understand. In this issue, we will attempt to address some of these puzzling questions.

In this way, several authors write on oxidative stress in the pathophysiology of target organ damage. G. Csányi and P. J. Pagano postulated that peptides mimicking the Nox4 B-loop and regions C-terminus on inhibit Nox4 activity and their findings suggest that Nox4 exists in a tightly assembled and active conformation which, unlike other Noxes, cannot be disrupted by conventional means. R. O. Marañon et al. demonstrated, in nonischemic 5/6 nephrectomized rat (NefR), a model of chronic kidney disease, that tempol improves NO-contents and basal tone, without decrease MAP, indicating that oxidative stress could be implicated early and independently to hypertension, in the vascular alterations. B. P. Campagnaro et al. proposed that the non-hemodynamic effects of angiotensin II are important for the damage observed in the two-kidney, one-clip (2K1C) renovascular hypertension model and demonstrated that 2K1C hypertensive mice have an elevated lymphocyte count, while undifferentiated bone marrow mononuclear cells counts were diminished.

N. Arias et al. describe mechanisms associated with inflammation and oxidative stress in the brain. N. Arias et al., in their study, examined the differential contribution of these leading factors to the oxidative metabolism of diverse brain limbic system regions frequently involved in memory process by histochemical labelling of cytochrome oxidase (COx). S.-L. Chan and G. L. Baumbach examined the role of genetic deficiency of NAD(P)H-oxidase subunit Nox2 in the function and structure of cerebral arterioles during hypertension and concluded that hypertension-induced superoxide production derived from Nox2-containing NADPH oxidase promotes hypertrophy and causes endothelial dysfunction in cerebral arterioles, possibly involving interaction with nitric oxide.

On the other hand, J. Alvarez-Camacho et al. describe the pathophysiologic link between the metabolic syndrome, obesity, hypertension, and adipokines at experimental and clinical level. J. Alvarez-Camacho et al. addresse topics related to modern aspects in the pathophysiology of hypertension such as the role of inflammation, environment, epigenetics, and adiponectin in hypertension, and Y. Mendizabal et al. discuss the central role of hypertension and vascular inflammation in metabolic syndrome and the role of sympathetic tone. Subsequently, they examine the link between endothelial dysfunction and insulin resistance. Finally, animal models used in the study of vascular function of metabolic syndrome are reviewed, in particular, the Zucker fatty rat and the spontaneously hypertensive obese rat (SHROB).

N. F. Renna et al. conducted a review of the mechanisms involved in the physiopathological vascular changes, adaptive and maladaptive, called vascular remodeling and the involvement of inflammatory mechanisms.

Finally, D. M. Babbitt et al. performed their analysis on a clinical protocol that demonstrated that aerobic exercise training might be a viable, nonpharmacological method to improve inflammation status and endothelial function and thereby contribute to risk reduction for cardiovascular disease in African Americans.

In conclusion, the observed elevation in inflammatory parameters in subjects who subsequently go on to develop hypertension is particularly relevant and creates options for potential primary prevention strategies. A number of therapeutic agents have been identified which are able to influence this inflammatory process and positively influence cardiovascular outcomes.

*Nicolas Federico Renna* *Nicolas Federico Renna*

